# Electroencephalographic Patterns in taVNS: A Systematic Review

**DOI:** 10.3390/biomedicines10092208

**Published:** 2022-09-06

**Authors:** Anna Carolyna L. Gianlorenco, Paulo S. de Melo, Anna Marduy, Angela Yun Kim, Chi Kyung Kim, Hyuk Choi, Jae-Jun Song, Felipe Fregni

**Affiliations:** 1 Department of Physical Therapy, Federal University of Sao Carlos, Sao Carlos 13565-090, Brazil; 2 Neuromodulation Center and Center for Clinical Research Learning, Spaulding Rehabilitation Hospital and Massachusetts General Hospital, Harvard Medical School, Boston, MA 02129, USA; 3 Medicine, Escola Bahiana de Medicina e Saúde Pública, Salvador 40290-000, Brazil; 4 União Metropolitana de Ensino e Cultura (UNIME) Salvador, Salvador 42700-000, Brazil; 5 Department of Otorhinolaryngology-Head and Neck Surgery, Korea University Medical Center, Seoul 08308, Korea; 6 Department of Neurology, Korea University Guro Hospital, Seoul 08308, Korea; 7 Department of Medical Sciences, Graduate School of Medicine, Korea University, Seoul 08308, Korea; 8 Neurive Co., Ltd., Gimhae 08308, Korea

**Keywords:** transcutaneous vagus nerve stimulation, taVNS, brain signals, EEG

## Abstract

Transcutaneous auricular vagus nerve stimulation (taVNS) is a newer delivery system using a non-invasive stimulation device placed at the ear. taVNS research is focused on clinical trials showing potential therapeutic benefits, however the neurophysiological effects of this stimulation on brain activity are still unclear. We propose a systematic review that aims to describe the effects of taVNS on EEG measures and identify taVNS parameters that can potentially lead to consistent EEG-mediated biomarkers for this therapy. A systematic literature review was carried out following the Preferred Reporting Items for Systematic Reviews and Meta-Analyzes (PRISMA) and the Cochrane handbook for systematic reviews. Clinical trials examining EEG parameters were considered, including absolute and relative power, coherence, degree of symmetry, evoked potentials, and peak frequency of all bands. According to our criteria, 18 studies (from 122 articles) were included. Our findings show a general trend towards increased EEG power spectrum activity in lower frequencies, and changes on early components of the ERP related to inhibitory tasks. This review suggests that quantitative electroencephalography can be used to assess the effects of taVNS on brain activity, however more studies are needed to systematically establish the specific effects and metrics that would reflect the non-invasive stimulation through the auricular branch of the vagus nerve.

## 1. Introduction

Due to its long path extending from its origin from the brainstem through the face and thorax down to the abdomen, the vagus nerve is also described as the “wandering nerve” [1]. The vagus nerve plays a widespread role maintaining autonomic tone among brain structures and peripheral organs. Traditionally, vagus nerve stimulation techniques have been developed to treat epilepsy and were first approved by the FDA in 1997 as an implantable treatment device [2]. Implantable vagus nerve stimulation is FDA-approved for therapeutic use in drug-resistant epilepsy and depression, and recent studies have shown promising results in treating various disorders such as cluster headache, heart failure, Alzheimer’s disease, anxiety disorder, obesity, sepsis, lung injury, rheumatoid arthritis, and diabetes [3,4]. However, this classical approach requires an invasive surgical procedure to the cervical vagus nerve trunk, and therefore is limited by the potential risks of surgical complications, cost, and accessibility.

Previous studies show evidence that the auricular branch of the vagus nerve, also known as the Alderman’s nerve or Arnold’s nerve, activates the nucleus tractus solitarii (NTS), the locus coeruleous (LC), the “classical” vagal projections, and has a demonstrated anti-inflammatory effect [5,6]. Functional magnetic resonance (fMRI) studies have suggested that afferent vagal nerve stimulation (VNS) may enhance GABA release through the NTS [5,7,8,9]. Moreover, Capone et al. demonstrated that taVNS increased the short-interval intracortical inhibition (SICI) in the right motor cortex. This specific transcranial magnetic stimulation metric is sensitive to GABAa activity [10]. Strong evidence has shown that taVNS also may affect the release of noreprinefrine in the brain. Recent fMRI studies demonstrated that the LC, a noradrenergic region, can be activated through the stimulation [5,7,8,9].

Transcutaneous auricular vagus nerve stimulation (taVNS) is a newer delivery system, using a stimulation device placed at the concha or tragus of the ear, which does not require surgery and therefore is more accessible to patients with demand [3]. Studies in the recent decade focus on the non-invasive method using non-implant taVNS as a safe and effective treatment of epilepsy, depression, schizophrenia, pain, migraine, Parkinson’s disease, tinnitus, impaired glucose tolerance, and induced atrial fibrillation [2,4,11].

The taVNS field is still in its infancy, and there is much to discuss for consideration in the parameters used. The current intensity is typically administered above the perceptual threshold, and below the pain threshold [12]. There have been several studies exploring the diverse “optimal” parameters for taVNS such as fMRI, heart rate variability, salivary alpha-amylase, pupillary responses, or P300 for norepinephrine release [13]. However, without a consensus on ideal parameters, taVNS research is predominantly focused on clinical trials showing potential therapeutic benefits for various pathologic conditions. Ideally, a biological marker, defined as being a substance, feature, or image that indicates the state of a system or an organism [14], would provide information that the auricular nerve was truly stimulated to affect biological processes causing an effect on intracranial structures. Without a reliable biomarker, we may not learn the best from the results of these studies with the best interpretation.

Electroencephalographic (EEG) findings have been widely discussed as possible biomarkers for the non-invasive brain stimulation (NIBS) field. EEG measurements are based on electrical potential differences between different electrodes on the scalp. A potential difference is caused by the propagation of the current flow induced by synchronized postsynaptic potentials in pyramidal neuron cell membranes. These measurements allow for the evaluation of effective connectivity between different cortical and subcortical regions and provide high temporal and spatial resolution for the analysis of brain stimulation effects, such as those of transcranial magnetic stimulation (TMS) and transcranial direct current stimulation (tDCS) [15,16]. Additionally, the use of EEG has been able to detect improvement of cortical connectivity after the use of NIBS such as tDCS in different disorders [17,18]. Thus, EEG has proven to be a useful tool in understanding the mechanisms behind NIBS and its effect on brain connectivity.

Based on that, electroencephalography is seen as a promising imaging tool to provide biomarkers for taVNS given it is a reliable, non-invasive, and inexpensive method to measure cortical activity [19]. Moreover, the effect of vagal nerve stimulation (VNS) has been associated with desynchronization and synchronization events recorded through EEG [20,21]. Significantly, these events have been associated with positive outcomes in conditions such as epilepsy [22]. This reinforces the potential for EEG to effectively demonstrate the effects of VNS in different neurological conditions.

Therefore, we propose a systematic review that aims to describe the effects of taVNS on EEG neurophysiological measures and identify taVNS parameters that can potentially lead to consistent EEG-mediated biomarkers for this therapy.

## 2. Methods

### 2.1. Literature Search and Study Selection

This review was registered at PROSPERO under the registration number 340328 and followed the guidelines of the Preferred Reporting Items for Systematic Reviews and Meta-Analyzes–PRISMA [23] and the Cochrane handbook for systematic reviews [24]. Literature search was performed in PubMed/Medline, Cochrane CENTRAL, LILACS, Embase and Web of Science databases until June 2022 using the following criteria for eligibility: (a) clinical trials using taVNS as intervention; (b) human population over the age of 18 years; (c) assessment of EEG data. All studies examining EEG parameters in humans were considered, including absolute and relative power, coherence, and degree of symmetry, evoked potentials (EP) and peak frequency of all bands. We therefore excluded the following articles: (a) lack of control group; (b) combination of taVNS with other intervention; (c) not taVNS (VNS or cervical VNS); and (d) abstracts without full text.

The search terms used were (Transcutaneous vagal nerve stimulation) OR (Transcutaneous vagus nerve stimulation) OR (Auricular vagal nerve stimulation) OR (Auricular vagus nerve stimulation) OR (tVNS) OR (taVNS) OR (aVNS) AND (EEG) OR (Electroencephalography) OR (Electroencephalogram). The references were extracted from the databases and imported to Endnote 20, which excluded the duplicates. All the references were further exported to Excel for manual evaluation. Titles and abstracts were screened independently by two authors (ACLG and PdSM) according to the criteria. Unclear references were assessed by the full text. After reaching a consensus about selected studies based on the inclusion and exclusion criteria, all full texts were retrieved for the data extraction and analyses.

### 2.2. Data Extraction

Two investigators (ACLG and PdSM) performed the data extraction on Excel supervised by a third senior investigator (FF). The researchers extracted and tabulated variables such as study design, population, age, sex proportions, and sample size; type of control, intervention, and taVNS stimulation parameters; adverse events; EEG parameters, results, and limitations of the studies.

### 2.3. Quality Assessment

Risk of bias was assessed in controlled studies (either parallel arm or cross-over RCTs) with The Cochrane Collaboration’s tool (RoB 2.0) [25] within each domain described by the instrument, concerning biases arising from the randomization process, deviations from intended interventions, missing outcome data, outcome measurement and selection of the reported results. Two authors (ACLG and PdSM) independently rated each randomized clinical trial (RCT) using this tool, and then they came to a consensus on each domain.

## 3. Results

### 3.1. Included Studies

**Figure 1:**
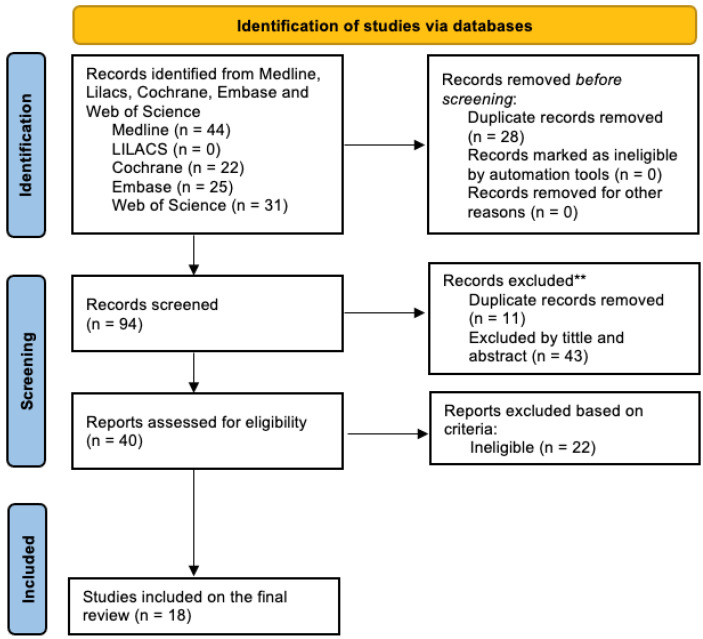
Flowchart of the included studies.

We identified 122 citations from all databases. After 28 duplicates were automatically removed, 94 titles and abstracts were filtered for relevance. From those, 11 duplicates were still found manually, and 43 articles were excluded based on title and abstract evaluating our eligibility criteria. The remaining 40 articles were evaluated in full text and five articles were excluded since they were poster abstracts with insufficient data presented. From the final 35 reports, 17 were found ineligible after accessing the full text. The final review included 18 studies (Figure 1).

### 3.2. Description of Included Studies

Across studies, the most frequent design was a crossover (*n* = 14; 77.78%) followed by parallel (*n* = 4; 22.22%). Almost all the studies included only healthy participants (*n* = 17; 94%), with a total number of 529 subjects, with a mean sample size of 28.05 subjects (range of 8–47), a mean participant age of 24.6 years, and with 45.37% of males included. The characteristics of each study and a description of the participants are further summarized in Table 1.

### 3.3. taVNS’ Stimulation Parameters

Regarding the parameters to perform taVNS, the majority of the studies only had one session of intervention (*n* = 16; 88.9%) with duration from 10 to 180 min with different devices, intensities, and frequencies. From all the studies reported in this review, the left ear and the left cymba conchae were the most stimulated regions (14 studies—77.78%). Regarding the control, all the studies had a sham group (18), and in 15 studies the earlobe was stimulated to simulate the active intervention’s sensation on the auricular branch of the vagus nerve. Regarding the specific parameters of stimulation, the ones more frequently used were frequency of 25 Hz in 14 studies (77.78%); pulse width in a range of 200–300 ms (77.78%); and adjustable intensity in 12 studies (66.67%). A detailed description of the taVNS in all the included studies can be found in Table 2.

### 3.4. Safety Data on taVNS

Only five of the included studies reported adverse events in their articles. No major adverse events during the period of these studies were reported. The cases reported varied from sensation of stimulation to skin irritation of the ear. The detailed description of the reported adverse events and safety data is present in Table 3.

### 3.5. Electroencephalography

The parameters of electroencephalography such as number of electrodes (4 to 257), the timing t it was recorded (pre-post or during) and sampling rate (250 to 5000 Hz) varied across studies. Furthermore, the type of task to record the ERP was very heterogeneous. We could detect a predominance in the use of different types of Oddball task, such as Bayesian and novelty tasks; the Go/No Go, different somatosensory and visual stimuli, Simon, learning and lexical recognition, action planning tasks, and readiness were also used. One study tested the taVNS effects on the transcranial magnetic stimulation potentials and another one on the heart-evoked potentials.

### 3.6. Effects of taVNS in Brain Oscillations

In our review, we detected the effects of taVNS in different EEG channels, waves, and metrics. There were five studies reporting its effects in the power spectrum activity, twelve studies in common ERP metrics, and one study with more unusual EEG measurement.

### 3.7. Power Analysis

Five of the included studies assessed different power spectrums in EEG activity (delta to beta), and some identified changes induced by taVNS, mainly in lower frequencies. Between them, Ricci et al. demonstrated a significant increase in delta oscillations when comparing the taVNS group pre and post stimulation, and when comparing the taVNS and the control group post stimulation in the frontocentral and central area mainly. Keute et al. measured brain oscillations during the Go-No Go task, and also found an increase in theta activity in the frontocentral area during conflict in comparison to sham.

Sharon et al., during resting, detected attenuations in higher frequency. In their study, they found a decrease in alpha in the active group after intervention, while there was no difference in the sham group. On the other hand, during the Eriksen Flanker task, Konjusha et al. performed a cluster-based analysis based on the RIDE decomposed data method, finding increases in alpha power in negative clusters at S-cluster, stimulus-related processes as perception and attention, C-cluster, reflection the stimulus response mapping process, and R-cluster, response-related processes as motor preparation and response execution, in central electrodes, while also finding decreases in alpha in positive clusters at S-cluster, C-cluster, and R-cluster in left hemisphere frontal electrodes in comparison to sham.

### 3.8. Effects of taVNS in Brain Event-Related Potentials

Most of the studies analyzed ERP metrics on this review. Among the common oscillation assessments during events we detected early events, such as P100 [34], P180 [33], P200 [27,34], P300 [28,29,33,36,40,42,43], and N200 [27,28,34,36], and late events, such as P600 [33], N400 [35], and N450 [33].

### 3.9. Early Events

#### 3.9.1. Positive Waves

Regarding the early positive waves, three studies measured P100, P200 or P180. Only one study measured P100 and found lower amplitudes at occipital electrodes in the taVNS group during visual stimulus of objects and food. This study also detected a smaller P200 amplitude in the taVNS group. However, regarding P200, Dumoulin et al. did not detect any effect of stimulation. Mertens et al. measured P180 and detected an increase in the wave in the right motor cortex after active taVNS during single-pulse measurements.

#### 3.9.2. Negative Waves

Our review detected seven studies that measured the effects of taVNS on negative waves during the tasks. However, the results are divergent. Pihlaja et al. detected a decrease in N200 peaks in the taVNS group mainly during the No Go task in frontal areas. However, Obst et al. found higher N200 amplitudes in central areas in the taVNS group during food and object food stimuli. Dumoulin et al. and Fisher et al. also measured N200 peaks during somatosensory stimuli and Simon tasks but did not find any differences.

### 3.10. Late Events

#### 3.10.1. Positive Waves

On the late positive waves, the most used metric of this review was P300. Although, it was not different in the taVNS groups among most of the reports [28,36,40,42,43], using different tasks to measure the ERPs. Noteworthy, Mertens et al., using TMS-ERPs, detected an increase in P300 in the right motor cortex after active taVNS and a widespread decrease after sham stimulation, both during the measure of SICI. Mertens et al. also reported P600 waves results and detected an increase in the right motor cortex after active stimulation.

#### 3.10.2. Negative Waves

Finally, the late negative waves were reported by only one study. Philips et al. analyzed the effects of stimulation in N400 peaks during passive and active learning tasks and lexical recognition in three different groups. Noteworthy, they detected a larger amplitude of N400 in the peristim and prime groups (different taVNS applications) in parietal sites during a lexical recognition task.

### 3.11. Brain Event-Related Potential Tasks Related with taVNS

An important piece of information retrieved by this review is in which type of task that the taVNS was able to modulate the brain oscillations, and which brain function they represent. In total, 14 studies used some specific tasks while recording EEG, however the tasks were very heterogeneous, and the event-related potential varied from somatosensory to learning processes.

#### 3.11.1. Power Spectrum Analysis

Two of these studies only measured the power spectrum differences between sham and active. Keute et al. performed the Go-No Go task, finding an increase in frontocentral theta activity in conflict during taVNS sessions in comparison to sham. Moreover, Konjusha et al. performed the Eriksen Flanker task, detecting an increase in alpha power in negative clusters in S, C, and R clusters in active stimulation.

#### 3.11.2. Brain Functions and the Event-Related Potentials

On the other hand, 12 studies measured the ERPs induced by taVNS using different types of tasks. The most common activity used to detect the ERPs was the Oddball task [40,42,43], and cognitive conflict resolution tasks [28,31,32,36].

#### 3.11.3. Visual/Attention ERPs

During tasks measuring attention, Ventura-Bort et al., Warren et al. 2020, and Warren et al. 2019 used the Oddball task to test the effect of taVNS in the P300 wave, not detecting any effect on the metric. Noteworthy, each of the studies used slightly different kinds of Oddball paradigms, Ventura-Bort et al. tested a novelty task and Warren et al. 2020 the Bayesian Oddball task, while Warren et al. 2019 used the classic and novelty paradigms.

Obst et al. used a different type of Oddball paradigm together with visual pictures of foods and objects. On this trial, they could detect differences elicit by taVNS, as lower P100 and P200 amplitudes in occipital electrodes and higher N200 amplitudes in the central area.

#### 3.11.4. Conflict Resolution/Control/Inhibition ERPs

During the Go-No Go task that measures control and inhibition, Pihlaja et al. was able to detect a main effect of taVNS in reducing N200 amplitudes in the frontal area, mainly in the No Go task. However, they did not detect any stimulation effect in P300.

Also measuring inhibition, Keute et al. 2018 were able to detect differences in the readiness potential for compatible trials between active and sham. They conveyed a marginally significant decrease in motor preparation in active stimulation after prime presentation during 280 and 380 ms. Fisher et al., also measured the taVNS effect on the N200 and P300 effect on the Simon task and found a reduction of N2 for incompatible trials following conflict (compared to following non-conflict) in the active taVNS group.

#### 3.11.5. Somatosensory ERPs

Dumoulin et al. tested the effect of taVNS in N200 and P200 during somatosensory stimuli (laser, cold, and vibrotactile sensation). However, the authors were not able to detect any differences between active and sham stimulation.

#### 3.11.6. Learning and Recognition ERPs

Phillips et al. tested the effect of taVNS on N400 during and before learning and recognition tasks. They conveyed that taVNS right before the task elicited higher amplitudes in central and parietal areas during passive learning. Furthermore, the authors detected larger amplitudes of N400 in parietal sites when stimulating during or right before the task in the lexical recognition task.

#### 3.11.7. Action Planning

During the action planning paradigm, Chen et al. analyzed the effects of taVNS in movement related potentials, finding only a difference between action and sham in the left-difficult task in the motor cortex. When analyzed, left-easy and right tasks were no different between groups.

### 3.12. Other Events

Mertens et al. used TMS-evoked potentials to analyze the effects of taVNS. They provided single-pulse and paired-pulse stimuli. In the single-pulse analysis in the region of interest, they could detect an increase in P180 in the right motor region after taVNS. During paired-pulse stimuli, on SICI, they conveyed a time and region of interest decrease in P300 and increase in N450 after sham stimulation, while they detected a region of interest increase in P300 and P600 in the same region after active stimulation.

Finally, Poppa et al. performed a different analysis of the brain waves related with the heart rate variability, called heart-evoked potentials (HEP). They divided the analysis by sensor and source levels, and in different clusters. On the sensor-level, they could detect lower HEP amplitudes on the left frontocentral area in cluster 1 and a greater HEP amplitude in centroparietal regions. On the source-level, they detected a bilateral greater magnitude on lateral and medial orbitofrontal cortex, anterior cingulate and subcallosal gyrus, and a left-lateralized effect on the operculum, postcentral gyrus, precentral gyrus, anterior and posterior insula, middle frontal gyrus, superior temporal gyrus, temporal pole, and anterior medial and temporal regions.

A detailed description of all EEG results is provided in Table 4.

## 4. Correlations between EEG Metrics and Clinical Data

Some studies explored correlations between the measured EEG metrics and clinical outcomes. Mertens et al. detected a correlation between change in resting motor threshold (rMT) and change in P180 amplitude after active taVNS (R^2^ = 0.632, *p* = 0.018). However, the results lost significance after Bonferroni correction. Additionally, Obst et al. detected a negative correlation between P200 amplitudes and BMI in both stimulation conditions (tVNS: r = −0.40, *p* = 0.028; sham: r = −0.41, *p* = 0.021). The amount of food intake after visual stimuli and stimulation also correlated with P200 amplitudes in the sham group (r = −0.38, *p* = 0.035) but not in the taVNS group. After being corrected for multiple testing with Bonferroni, all correlations also lost significance, besides being supported by the corresponding Bayes Factors (BMI and P200: tVNS = 2.27, sham = 2.85; food intake and P200: tVNS = 0.7, sham = 2.27). Sharon et al. detected a correlation between the differences in alpha attenuation (tVNS vs sham conditions) and the differences in applied current (tVNS vs sham conditions; r = 0.49, *p* = 0.02). Therefore, stronger sham stimulation showed less difference in alpha attenuation.

Finally, Ventura-Bort et al. found a correlation between some “autonomic measures” and “electrophysiological recording”. They detected that enlarged P300b amplitudes were correlated with an increase in salivary alpha amylase (sAA) post stimulation only for the easy target condition (r = 0.56, *p* = 0.025), while this association was not observed during sham stimulation. These findings may indicate a potential association of noradrenergic activation and the P300b, which may be prevalent under vagal stimulation.

## 5. Quality Assessment and Risk of Bias

**Figure 2:**
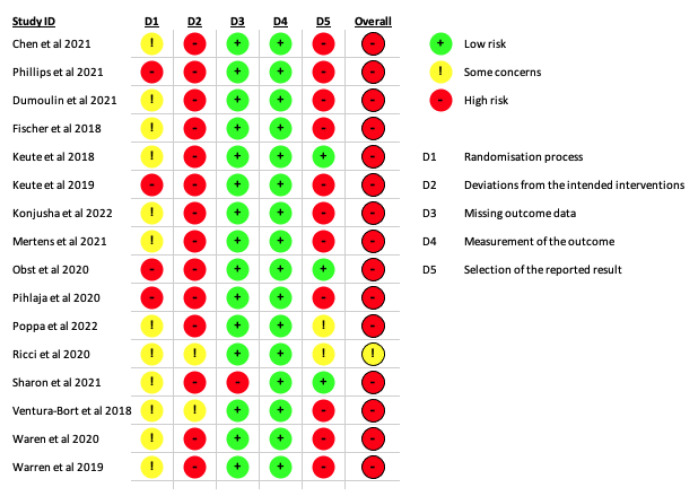
Risk of bias of the included studies.

In this review, most of the studies were evaluated as a high risk of bias. The domains where we detected major issues were in the deviation from the intended interventions (D2), that most of the studies did not report the number of individuals analysed after randomization or did not stand that they performed an intention-to-treat analysis, and the selection of the reported result (D5), since almost all the studies provided multiples outcomes and different types of analysis, increasing the probability of type one error. The domain that measures the randomization process (D1) also presented some issues, as most of the studies were classified as “some concerns” and the rest as high risk of bias. In this domain, some of the studies did not perform or mention the randomization, did not describe the main baseline variables between the groups, or did not perform or mention about the allocation concealment (Figure 2).

## 6. Discussion

In this systematic review we aimed to determine EEG patterns in the presence of taVNS. Our findings show that there is a general trend towards increased EEG power spectrum activity in lower frequencies, i.e., delta and theta frequency band, and changes on early components of the ERP related to inhibitory and conflict resolution tasks. On the other hand, most of the studies that assessed P3 showed no changes. However, because of EEG’s protocol heterogeneity preventing a meta-analysis, our findings are based on a qualitative examination.

### 6.1. taVNS Effects on Typical Frequency Bands

The classical EEG resting analysis divides oscillations into specific frequency bands. For example, in adults, typical frequency bands and their approximate spectral boundaries are delta (1–4 Hz), theta (4–8 Hz), alpha (8–12 Hz), beta (13–30 Hz), and gamma (30–100 Hz) bands. Ricci et al., using power spectrum EEG analysis to assess the effects of taVNS on cerebral cortex activity, found that delta activity was significantly higher after active taVNS while showing no changes after sham stimulation. Delta waves, which originate in the cortical layer, are the most significant EEG feature of human non-rapid eye movement sleep. Delta waves are essentially non-existent in the physiological state during waking, but they are prominent when a subcortical brain injury develops [44,45]. Non-lesional delta waves, on the other hand, are thought to arise from a greater number of synchronous oscillating neurons or from increased activity of such neurons. These findings support the theory that delta oscillations in the human brain are generated by subcortical deep generators, which may be impacted by brainstem nuclei activity. As a result, the increase in delta activity reported following taVNS could be explained by subcortical activation of vagus nerve-related brainstem regions. The tractus solitarius nucleus projects to the locus coeruleus and the raphe nuclei, providing broad serotonergic innervation to the neocortex. Therefore, the rise in delta activity could be an unintended consequence of the activation of inhibitory subcortical pathways [38].

Keute et al. (2019) assessed theta activity on the frontal midline because a transient spectral power increase in the theta band over frontocentral electrodes is an established marker for control and adaptation processes: It can be seen in reaction to novel stimuli, response conflicts, errors, and other events that may necessitate behavioral adaptation [46]. In this study, taVNS improved accuracy across situations and lowered the costs of go/change response conflicts in terms of performance, and there was a transitory frontocentral increase of theta activity to target stimuli in EEG data, which was amplified in conflict.

Regarding the alpha frequency band, its oscillations are frequent during wakefulness when there is a dissociation from the sensory environment, and it is thought to indicate an “idling” state of cortical activity or inhibition of task-irrelevant cortical areas [47] that is anticorrelated with arousal-promoting activity such as the LC–NE system. During resting EEG, Sharon et al. found that short tVNS pulses induce alpha attenuation to a greater extent than the sham stimulation. According to the authors, these support the hypothesis that tVNS could activate endogenous arousal-promoting neuromodulatory signaling such as LC–NE activity, as is known to occur in invasive VNS [48].

During the Eriksen Flanker task, Konjusha et al. found that alpha-frequency band activity revealed modulatory effects at all investigated coding levels as revealed by RIDE of the EEG alpha signal. Their results showed that alpha-band activity was lower in prefrontal regions during taVNS stimulation and lower when there was also a decline in task performance. Alpha-band activity is linked to attentional processing and cognitive control mechanisms [49] in that they are relevant for the suppression of irrelevant/interfering information [47]. Mainly prefrontal regions are critically involved in such top-down control processes [50]. It thus seems that taVNS has reduced the property of alpha-band activity to suppress the interfering effects of irrelevant information in prefrontal cortices.

### 6.2. taVNS Effects on ERP Metrics

EEG and the study of ERPs allow examining brain activity as a direct result of a specific sensory, cognitive, or motor event. They are supposed to represent the summed activity of postsynaptic potentials created when many cortical pyramidal neurons (in the hundreds or millions) fire in synchrony while processing information [51]. Human ERPs can be classified into two groups. The early waves are referred to as ‘sensory’ or ‘exogenous,’ because they are highly dependent on the stimulus’s physical properties. Later ERPs, on the other hand, indicate how the individual analyzes the stimulus and are referred to as ‘cognitive’ or ‘endogenous’ ERPs since they investigate information processing [52].

On early waves, Obst et al. showed that TaVNS significantly decreased P1 and P2 amplitudes and increased N2 amplitudes compared to sham stimulation. However, they found no taVNS-dependent modulation of food intake nor a specific food-related stimulation effect on the ERPs. The authors suggest that there is a general effect of TaVNS on those ERP components, indicating a possible influence on attentional and inhibitory aspects in visual perception processes [34]. Mertens et al. in their study using TMS, showed an increase in P180 and a decrease in P60, demonstrating that taVNS affected both cortical and corticospinal measures of excitability in subjects who tolerated high stimulation currents. The results of this sub-analysis imply that in order to significantly activate the afferent fibers and important targets of the vagus nerve trajectory and cause neuromodulatory effects, a high stimulation current intensity may be necessary [33].

Pihjala et al. observed a change in N2 ERP, considered a neural marker of cognitive control. In comparison to sham stimulation, the peak amplitude of frontal NoGo-N2 was significantly lower after taVNS. Reduced NoGo-N2 amplitude combined with unaffected response inhibition shows that during taVNS, fewer cognitive control resources were required to avoid reacting [36].

Most taVNS-EEG studies focused on the parietally distributed P3, presumably due to the suggested shared links with NE. The P3b is thought to be affected by the LC-NE system [28,40]. Furthermore, links between phasic pupil dilations, an indicator of LC activity and P3 amplitudes, have been reported [43]. P3 amplitude is defined as the largest positive peak of the ERP waveform within the time window of 300–500 ms, and the corresponding latency is defined as the time interval from stimulus onset to the point of maximum positive amplitude within the same time window [53]. P3 potentials can reflect various cognition-related brain functions, such as attention allocations and working memory. Thus, the P3 can be regarded as a potential biomarker to evaluate a subject’s processing capacity in an experimental task.

However, most of the studies showed no changes on P3 using different tasks to measure the ERPs: classical Oddball task and novelty Oddball [40,42,43], Bayesian Oddball task [42], Go/NoGo task [36] and Simon task [28]. Previous studies on EEG showed that larger P3 was found for conflict compared to non-conflict trials and a sequential modulation of the P3 amplitude, indicating that the P3 amplitude during conflict monitoring seems to reflect the degree of conflict to be resolved. The P3 during conflict monitoring seems to be associated with the allocation of the attentional resources to the current stimulus and more linked to the P3a, rather than to the P3b [54]. Different neurotransmitter systems are involved in generating the P3a and P3b. Whereas the NE system is related to the P3b, the P3a seems to be mediated by the dopaminergic system, which is probably the reason why taVNS did not impact the P3(a) on these studies. Other studies also suggested that the traditional Oddball task is not sensitive enough to render the brain stimulation effects of taVNS detectable.

### 6.3. Limitations

Across the studies included, while many of the parameters (frequency, pulse width and currency intensity) have been used with standard definitions, often 25 Hz, between 200–300 ms, and current intensity typically administered between perceptual and pain threshold. However, terms such as “duration” are inconsistent over papers to refer to different scales of time, from a few minutes to some hours. Without investigating ideal parameters, taVNS researchers carried on often using parameters similar to those administered in cervically implanted VNS analogs. Based on that, new studies should focus on assessing the effects of different dosing metrics to establish the best parameters of stimulation.

One of the sources of heterogeneity between the studies was the use of different tasks to measure the event-related potentials. A commonly used paradigm in the literature is the Oddball task, and during our search we detected three studies using the paradigm [40,42,43]. However, even though these studies differ in the subgroup of the Oddball task used, Ventura-Bort et al. assessed the novelty Oddball, while Waren et al. assessed the Bayesian Oddball in 2020, and the novelty and classic Oddball in 2019. Other types of events were used less frequently, such as the Go-No Go, somatosensory stimuli, Simon task, visual stimuli, TMS, learning task and lexical recognition, action planning task, experimental task to measure the readiness potential, and heart-evoked potentials. This variability makes it hard to pool the data and make a fair comparison between the effects of taVNS in each study. We hope that this review and future research are going to help to find more specific tasks and metrics to measure the effects of taVNS.

Furthermore, from the 16 studies, 14 had to control the same stimulation at the earlobe, which is known to be relatively free of vagal innervation. Electrical stimulation of the earlobes has long been used as part of a non-invasive transcutaneous brain stimulation technique known as cranial electrotherapy stimulation (CES), which is approved by the Food and Drug Administration (FDA) for the treatment of insomnia, depression, and anxiety [55,56]. The earlobe is primarily innervated by the great auricular nerve that originates from the cervical nerve (C2); thus, CES does not stimulate the ABVN [57]. Although its working mechanisms are not yet fully understood, CES is thought to modulate several brain areas. fMRI analyses have revealed that CES results in negative BOLD changes (deactivation) in the precuneus, precentral and postcentral gyri, posterior cingulate gyrus, and occipital cortex [58]. These areas were also affected by tVNS in fMRI studies, and therefore, earlobe stimulation could induce BOLD changes in the limbic system and other areas that are similar to those observed in response to tVNS, but the degree of activation seems to be much weaker than that induced by tVNS.

As a general limitation of our review, even though our data show some patterns of brain activity in healthy individuals under TaVNS, the included studies were quite diverse, preventing a meta-analysis. Also, our findings are based on a qualitative examination of the research. The literature usually does not provide the mathematical values of the EEG metrics, sometimes only providing statistical metrics (as *p* values and F values), which makes the process difficult to pool and synthesize the data. We strongly suggest that the next studies provide the full information about the metrics analyzed, as means and standard deviations, or any association metric. Finally, all studies available measuring taVNS effects in quantitative EEG only performed one session of stimulation and measured the EEG in varied periods after that. This is a main limitation of the literature, since when stimulating the nervous system, we would expect online acute changes in neurophysiological markers or long-lasting effects due to neuroplasticity. The last one, based on older techniques, such as tDCS and TMS, requires more sessions to be induced [59,60]. One possible problem is that when measuring the effects of taVNS sometime after one session, we could lose the potential effects of the intervention in the brain waves.

## 7. Conclusions

We aimed to synthesize the evidence of the effects of taVNS in the electroencephalography. Our review shows preliminary information that taVNS could influence cortical activity, mainly showing a trend to increased EEG power spectrum activity in lower frequencies (delta and theta), divergent results in higher frequencies (alpha), and changes on early components of the ERP related to inhibitory tasks with no P3 changes. However, there was a high heterogeneity between the studies, being hard to compare the results, and in the future, more homogeneous, bigger, longer, and well-designed studies are needed to make stronger conclusions about the effects of taVNS in brain activity measured by EEG.

## Figures and Tables

**Table 1:** Descriptive data of the included studies.

Author	Design	Population	Age (M, SD or Range)	Sex	Sample Size (Active/Control)
Chen et al., 2021 [26]	Parallel	healthy	23.4; 1.7	13F/15M	14/14
Dumoulin et al., 2021 [27]	Cross-over	healthy	A: 27.32; 9.11 B: 30.13; 11.23	12F/10M 6F/9M	22/22 15/15
Fisher et al., 2018 [28]	Cross-over	healthy	20.3; 1.4	18F/3M	21/21
Gadeyne et al., 2022 [29]	Cross-over	healthy	21.23; 1.63	21F/18M	39/39
Keute et al., 2020 [30]	Cross-over	healthy	23.8; 21–28	16F/6M	22/22
Keute et al., 2018 [31]	Cross-over	healthy	25.1; 2.4	8F/8M	16
Konjusha et al., 2022 [32]	Cross-over	healthy	23.57; 0.51	37F/8M	45/45
Mertens et al., 2021 [33]	Cross-over	healthy	22–32	15M	15/15
Obst et al., 2020 [34]	Cross-over	healthy	23; 2	15F/16M	31/31
Phillips et al., 2021 [35]	Parallel	healthy	Priming: 22.7; 4.19 Peristim: 21.7; 2.87 Sham: 22.1; 4.01	8F/4M 9F/4M 12F/8M	12 13 20
Pihlaja et al., 2020 [36]	Cross-over	healthy	25.5; 4.8	16F/9M	25/25
Poppa et al., 2022 [37]	Cross-over	healthy	23.1; 5.01	27F/18M	45/45
Ricci et al., 2020 [38]	Cross-over	healthy	30.5; 6.02	8M	8/8
Sharon et al., 2021 [39]	Cross-over	healthy	28.08; 5.84	24M	25/25
Ventura-Bort et al., 2018 [40]	Cross-over	healthy	20.3; 1.4	18F/3M	21/21
Yifei et al., 2022 [41]	Parallel	Disorder of consciousness	38.08; 9.38	12M	6/6
Warren et al., 2020 [42]	Cross-over	healthy	20.55; 2.18	34 F/8M	47/47
Warren et al., 2019 [43]	Cross-over	healthy	A: 22.6 B: 23.6 C: 22.1	28F/6M 9F/11M 17M	24/24 20/20 17/17

M: mean or median; SD: standard deviation.

**Table 2:** Description of stimulation parameters.

Author	Intervention	Control	N of Sessions	taVNS Parameters	Duration	Device	Site
Chen et al., 2021 [26]	taVNS	Sham in earlobe	1	25 Hz, 0.5 mA, 200–300	30 min prior the session and continued throughout the entire session2	(CM02, Cerbomed, Erlangen, Germany)	left cymba conchae
Dumoulin et al., 2021 [27]	taVNS	Sham in earlobe	1	25 Hz, adjustable intensity (to elicit a maximal, but non-painful, tingling sensation), 250, 30 s ON/30 s OFF	180 m	Nemos/Vitos, Erlangen, Germany	left cymba conchae
Fisher et al., 2018 [28]	taVNS	Sham in earlobe	1	25 Hz, adjustable intensity (above the detection threshold and below the pain threshold 1.49 ± 1.3 mA), 200–300	36 m	Medical CMO2, Cerbomed, Erlangen, Germany	left cymba conchae
Gadeyne et al., 2022 [29]	taVNS	Sham in earlobe	1	25 Hz, adjustable intensity (above the detection threshold and below the pain threshold mean 0.6 ± 0.3 mA), 250; 7 s/18 s on-off	17 m during the task	Medical Nemos, Cerbomed, Germany	left cymba conchae
Keute et al., 2020 [30]	taVNS	Sham in earlobe	1	25 Hz, adjustable intensity (máx 3 mA 2.6/2.37 ± 0.16 mA), 200; 30 s/30 s on–off	76 m	Medical Digitimer DS7 (Designed for Human Research Use—NOT a medical)	left cymba conchae
Keute et al., 2018 [31]	taVNS	Sham in earlobe	1	25 Hz, adjustable intensity (Stimulation intensity was set to 8 mA, if tolerable for the subject, and else individually adjusted below pain threshold. 7.5/5.9), 200; 30 s/30 s on–off	25 m prior task	Medical Digitimer DS7 (Designed for Human Research Use—NOT a medical)	left cymba conchae
Konjusha et al., 2022 [32]	taVNS	Sham in earlobe	1	25 Hz, 0.5 mA, 200–300, 30 s/30 s on–off	20 m prior	Cerbomed atVNS device	left outer ear
Mertens et al., 2021 [33]	taVNS	Sham in earlobe	1	25 Hz, adjustable intensity (gradually increased until the participant could perceive the stimulation, but remained 0.1 mA below the pain threshold, 7 s on,18 s off, 250	60 m	Medical Nemos, Cerbomed, Germany	left cymba conchae
Obst et al., 2020 [34]	taVNS	Sham in the outer upper ear	1	25 Hz, 0.6 mA, 30 s on off	120 m	Medical Nemos, Cerbomed, Germany	left cymba conchae
Phillips et al., 2021 [35]	taVNS priming taVNS peristim	Sham (receive taVNS outside of calibration and ramping)	2	300 Hz, adjustable intensity (0.2 mA below their perceptual threshold), 50	10 min prior or immediately preceding	Digitimer DS8R Biphasic Constant Current Stimulator	left outer ear
Pihlaja et al., 2020 [36]	taVNS	Sham in earlobe	1	30 Hz, adjustable intensity (1.6 mA and 3.2 mA), 250		CE approved tVNS device (Salustim Group, Kempele, Finland)	Left inner tragus
Poppa et al., 2022 [37]	taVNS	Sham in earlobe	1	25 Hz, adjustable intensity (The intensity was slowly increased from 0.1 mA in increments of 0.1 mA until the participant first detected a tingling sensation, recorded as the perceptual threshold. The intensity was increased in 0.1 mA increments until the sensation was reported to be unpleasant or pricking (exciting Ad fibers). This procedure was repeated three times. The average of the detection and pain thresholds was set as the stimulation intensity), 7 s on and 18 s off, 250	15 min	NEMOS^®^ device	left cymba conchae
Ricci et al., 2020 [38]	taVNS	Sham in earlobe	1	30 Hz, up to 8 mA (above the detection threshold and below pain perception), 500	60 min	Twister-EBM (medical but other nerves)	left inner tragus
Sharon et al., 2021 [39]	taVNS	Sham in earlobe	1	25 Hz, adjustable intensity (to a level experienced as just below painful, adjusted for each participant), 200–300		Medical Nemos	left cymba conchae
Ventura-Bort et al., 2018 [40]	taVNS	Sham in earlobe	1	25 Hz, adjustable intensity (above the detection threshold and below the pain threshold 1.49/1.3 mA), 200–300, continuous.	28 m	CMO2, Medical Cerbomed, Erlangen, Germany	left cymba conchae
Yifei et al., 2022 [41]	taVNS	Sham in the tail of helix	28	20 Hz, 4–6 mA, >1 ms wave width	30 min	The Huatuo brand electronic acupuncture instrument (SDZ-II_B type, Suzhou Medical Products Factory Co., Ltd.).	Bilateral auricular concha
Warren et al., 2020 [42]	taVNS	Sham in earlobe	1	25 Hz, 0.5 mA, 200–300		NEMOS^®^ taVNS	left cymba conchae
Warren et al., 2019 [43]	taVNS	Sham in earlobe	1	25 Hz, 0.5 mA, 200–300	20 min before than continue with the task	NEMOS^®^ taVNS	left cymba conchae

**Table 3:** Reported adverse events of taVNS.

Author *	Safety—Adverse Effects
Fisher et al., 2018 [28]	Subjective ratings indicated that the side effects of the stimulation were minimal, and no differences between stimulation conditions, except for the physical subjective experience of the stimulation, with higher ratings for the tVNS condition
Konjusha et al., 2022 [32]	No differences and blinding successful
Ricci et al., 2020 [38]	No major adverse events were registered during the experimental sessions
Sharon et al., 2021 [39]	Sham and tVNS conditions did not differ in any of the parameters of subjective averseness examined.
Ventura-Bort et al., 2018 [40]	The side effects of the stimulation were minimal, no differences between stimulation conditions, except for the sensory experience of the stimulation, with higher ratings in the tVNS condition, sensation under the electrodes; skin irritation in the ear, compared to sham. These results indicate that no unpleasant side-effects were experienced in either of the two conditions.

* The other studies did not report any adverse event.

**Table 4:** EEG findings of the included studies.

Author	Electrodes (Total)	Sampling Rate (Hz)	EEG Modality	Main EEG Outcomes	Results	Limitations
**Power analysis**						
Ricci et al., 2020 [38]	32	5000	Resting pre and post stimulation	Microstates and power spectrum	**Microstates**: Global explained variance: no significant difference was found. Templates: Only microstate A: a significant increase in mean duration in the active group (69.1 [67.8–75.2] ms for Pre. vs. 74.6 [68.4–77.5] ms for Post., *p* = 0.03, effect size (r) = 0.58), and a significant difference post stimulation (74.6 [68.4–77.5] ms for active and 64.1 [63.4–67.3] ms for Sham; *p* = 0.02). **Power spectrum**: no differences in theta, alpha, and beta frequencies. Delta power revealed significant increasing in several EEG channels (FZ, FcZ, Cz, F4, FC2, FC6, C4, CP2, CP6, P4, P8, C3, FC1, CP1; *p* < 0.01) for active pre vs. active post and for active post vs. sham post (FcZ, FC1, FC2, Cz, C4, CP2; *p* < 0.05)	Exploratory study, multiple comparisons, small sample, short resting state conditions.
Sharon et al., 2021 [39]	256	-	Resting during taVNS	Attenuation in alpha oscillations	Active group: attenuation of alpha in comparison to baseline (mean, 94.35% ± 2.2% of baseline, *p* = 0.003). Sham group: No attenuation from baseline (mean, 103.55 ± 2.4% of baseline)	Only male subjects, small sample
Yifei et al., 2022 [41]	62	2500	Resting state	Power differences and coherence analysis	No differences between active and sham groups were detected after stimulation.	Not clear representation of the results and analysis, small sample size, short follow-up.
Keute et al., 2020 [30]	64	500	ERP during Go/No Go task	Power differences	**Cue-locked**: no differences between active and sham **Target-locked**: increase in frontocentral theta activity (*p* < 0.029), time-averaged frontal midline (200–600 ms post-target) in conflict (stop and change) trials increased in tVNS sessions by 0.4 dB compared with sham sessions (χ2 = 4.3, *p* = 0.039).	No pre-specified outcomes, ceiling effects
Konjusha et al., 2022 [32]	60	-	ERP during Eriksen Flanker task	Power differences for theta and alpha using cluster-based permutation task	**Theta**: No differences of cluster-based permutation task modulation between active and sham. **Alpha**: CPTs revealed significant differences for S-, C- and R-cluster (*p* < 0.048). A negative cluster suggests that the alpha power was larger in the active stimulation condition than in the sham condition, whereas a positive cluster suggests smaller alpha power in the active condition. **S-cluster**: Significant alpha-band increasing were found as indicated by a negative cluster of central electrodes (Cz, FCz, FC1, CP1, F1, FC2, CP2, CPz, FC4; *p* = 0.007) and decreasing in a positive cluster at left hemisphere frontal electrodes (F5, Fp1, AF7, FT7, T7, FT9; *p* = 0.026). **C-cluster**: Significant alpha-band increasing modulations could be shown, as indicated by a negative cluster of central electrodes (Cz, FC1, FC2, CP2; *p* = 0.041) and a decreasing in positive cluster at left hemisphere frontal electrodes (F5, Fp1, AF7, FT7, FT9; *p* = 0.044). **R-cluster**: Significant alpha-band power increasing were also found for the R-cluster, as indicated by a negative cluster of central electrodes (Cz, FC1, FC2, CP2; *p* = 0.040) and a decreasing positive cluster at left hemisphere frontal electrodes (F5, Fp1, AF7, FT7, FT9; *p* = 0.033).	No pre-specified outcomes, multiple comparisons.
**ERP’s metrics**						
Ventura-Bort et al., 2018 [40]	257	250	ERP during novelty Oddball task	Effects on P300b amplitudes	**P300b**: No significant changes	Small sample and gender imbalance
Warren et al., 2020 [42]	32	256	ERP during Bayesian Oddball task	Effects on P300 amplitude	**P300**: No significant changes	Gender imbalance
Warren et al., 2019 [43]	64	512	ERP during Oddball and novelty Oddball tasks	Effects on P300 amplitude	**P300**: No significant changes in both tasks and experiments	Fixed stimulation intensity of 0.5 mA and relatively small sample size
Gadeyne et al., 2022 [29]	26	1024	Auditory Oddball paradigm	Effects on P300b	No significant differences between groups	Lack of inclusion of additional methods of measurement
Pihlaja et al., 2020 [36]	64	500	ERP Go/No Go task	Frontal N200 and centro-parietal P300	**N200**: main effect of stimulation status (tVNS, Sham, F [1, 17] = 14, 41, *p* = 0.001) and interaction of trial type (Go vs. NoGo) and stimulation status (F [1, 17] = 5.06, *p* = 0.038) reducing N200 amplitudes in frontal area. When analyzed separately, the stimulation status had an effect in the No Go, but not in the Go task. **P300**: no significant effects of stimulation.	Stimulation artifact mainly in reference electrodes, small sample size.
Dumoulin et al., 2021 [27]	32	1000	ERP during somatosensory stimuli	Laser-evoked ERPs analyzed through N200 and P200	**Laser-evoked ERPs, cool-evoked ERPs, and vibrotactile-evoked ERPs:** **N200**: no significant effect of condition. **P200**: no significant effect of condition.	Low sign to noise ratio, small sample size, lack of statistical power, use of earlobe as sham condition.
Fisher et al., 2018 [28]	257	250	ERP during Simon task	N200 and P300 amplitudes	**N200**: no significant stimulation effects. However, reduction of N2 for incompatible trials following conflict (compared to following non-conflict) in taVNS. **P300**: no significant stimulation effects.	Lack of pre-specified outcomes, small sample size, multiple comparisons.
Obst et al., 2020 [34]	29	-	ERP during visual stimuli of objects and food items (similar to the Oddball task)	Differential effect on ERPs to food vs. object pictures on N100, P100, N200, P200, P300, and LPP.	**P100**: lower amplitudes in the tVNS (F [1,30] = 5.36, *p* = 0.028) at occipital electrodes. **P200**: smaller P2 amplitude in the tVNS (*p* = 0.018) in occipital area **N200**: higher N2 amplitude in the tVNS (*p* = 0.012) in central area.	Small sample size, multiple comparisons.
Mertens et al., 2021 [33]	64	5000	TMS evoked potentials	Pre-post changes in P3, N45, P6, N100, and P180	**Single-pulse: ** ***Time window of interest:*** no significant changes ***Region of interest:* P180**: increase in the right motor region after active taVNS (*p* = 0.018) SICI: ***Time window of interest:*** Sham taVNS: **P300**: widespread decrease after sham taVNS (*p* = 0.013) **N450**: widespread increase after sham taVNS (*p* = 0.008) ***Region of interest:*** **Active taVNS: P300**: increase in the right motor region after taVNS (*p* = 0.016) **P600**: increase in the right motor region after taVNS (*p* = 0.019) **Sham taVNS: P300**: widespread decrease after sham taVNS (*p* = 0.008) **N450**: widespread increase after sham taVNS (*p* = 0.003) **LICI: ** No significant differences were found.	No pre-specified outcomes, multiple comparisons, small sample size, not register the perceptions taVNS threshold, not include sham-TMS measurements.
Phillips et al., 2021 [35]	64	1000	ERP during passive and active learning tasks, and lexical recognition test	N400 amplitude and topography	**N400: ** **Passive word learning task**: **Amplitude**: peristim and sham group presented larger amplitude over central and parietal. (*p* ≥ 0.001) **Topography**: negativity centered over central or centro-parietal midline sites with exception of priming group (central, frontal, and frontal polar sites). **Active word learning task**: No effects of stimulation were found. **Lexical recognition test:** **Amplitude**: larger effect amplitude at parietal sites for peristim (*p* = 0.002) and priming (*p* = 0.01). **Topography**: broad centro-parietal negativity for all taVNS groups and larger amplitude for peristim and sham.	Small sample size and multiple testing.
Chen et al., 2021 [26]	64	1000	ERP during the action planning paradigm	Difference in movement-related cortical potentials amplitude differences in LD/LE/RD/RE	Significant difference was observed between active group and sham group especially in Left-difficult (LE) task (*p* = 0.004) in the motor cortex.	Stimulation set at 0.5 mA, sham in the earlobe.
Keute et al., 2018 [31]	4	1000	ERP during experimental task	Modulation of negative compatibility effect and lateralized readiness potentials components	**Readiness potential difference**: **For compatible trials**: significant decrease in cortical motor preparation in active taVNS in the time window from 280 to 380 ms after prime presentation (*p* = 0.049) in motor cortex. **For incompatible trials**: no difference between active and sham	Small sample size.
*Other metrics*						
Poppa et al., 2022 [37]	64	-	Resting pre, during, and post stimulation	Heart-evoked potentials	**Sensor-level**: **Cluster 1**: lower HEP voltage amplitudes on left frontocentral. **Cluster 2**: centroparietal regions and greater HEP in centroparietal regions. **Source-level:** **Cluster 1**: greater magnitude observed bilaterally in lateral and medial sectors of the orbitofrontal cortex, anterior cingulate and subcallosal gyri. Left-lateralized effect on the operculum, postcentral gyrus, precentral gyrus, anterior and posterior insula, middle frontal gyrus, superior temporal gyrus, temporal pole, and anterior medial temporal regions.	Low spatial precision.

## Data Availability

Not applicable.
